# Epigenotyping in Peripheral Blood Cell DNA and Breast Cancer Risk: A Proof of Principle Study

**DOI:** 10.1371/journal.pone.0002656

**Published:** 2008-07-16

**Authors:** Martin Widschwendter, Sophia Apostolidou, Elke Raum, Dietrich Rothenbacher, Heidi Fiegl, Usha Menon, Christa Stegmaier, Ian J. Jacobs, Hermann Brenner

**Affiliations:** 1 Department of Gynecological Oncology, UCL Institute for Women's Health, University College London, London, United Kingdom; 2 Division for Clinical Epidemiology and Ageing Research, German Cancer Research Center, Heidelberg, Germany; 3 Saarland Cancer Registry, Saarbrücken, Germany; Dalhousie University, Canada

## Abstract

**Background:**

Epigenetic changes are emerging as one of the most important events in carcinogenesis. Two alterations in the pattern of DNA methylation in breast cancer (BC) have been previously reported; active estrogen receptor-*α* (ER-*α*) is associated with decreased methylation of ER-*α* target (ERT) genes, and polycomb group target (PCGT) genes are more likely than other genes to have promoter DNA hypermethylation in cancer. However, whether DNA methylation in normal unrelated cells is associated with BC risk and whether these imprints can be related to factors which can be modified by the environment, is unclear.

**Methodology/Principal Findings:**

Using quantitative methylation analysis in a case-control study (n = 1,083) we found that DNA methylation of peripheral blood cell DNA provides good prediction of BC risk. We also report that invasive ductal and invasive lobular BC is characterized by two different sets of genes, the latter particular by genes involved in the differentiation of the mesenchyme (PITX2, TITF1, GDNF and MYOD1). Finally we demonstrate that only ERT genes predict ER positive BC; lack of peripheral blood cell DNA methylation of ZNF217 predicted BC independent of age and family history (odds ratio 1.49; 95% confidence interval 1.12–1.97; P = 0.006) and was associated with ER-*α* bioactivity in the corresponding serum.

**Conclusion/Significance:**

This first large-scale epigenotyping study demonstrates that DNA methylation may serve as a link between the environment and the genome. Factors that can be modulated by the environment (like estrogens) leave an imprint in the DNA of cells that are unrelated to the target organ and indicate the predisposition to develop a cancer. Further research will need to demonstrate whether DNA methylation profiles will be able to serve as a new tool to predict the risk of developing chronic diseases with sufficient accuracy to guide preventive measures.

## Introduction

Each year, more than 1.15 million new cases of breast cancer are diagnosed worldwide [1]. Currently the Gail model, based on epidemiological risk factors, is the best we have of predicting overall risk of invasive breast cancer and the concordance statistics for correctly classifying women with respect to this outcome is 58–59% [2].

Epigenetic changes, in particular DNA methylation, are emerging as one of the most important events in carcinogenesis [3]–[6]. Recently, an increasing body of evidence from animal studies demonstrated that environmental factors can result in epigenetic modifications and subsequent changes in the risk of developing disease [7], [8]. There is preliminary evidence from small studies that peripheral blood cell DNA contains epigenetic information, which is a valuable predictive marker of an individual's risk of developing cancer [9], [10], [11]. Only the largest of these studies, involving 172 individuals and analyzing one locus, demonstrated that epigenetic alterations are able to predict the risk of colorectal cancer independently of family history [9].

We have undertaken a large scale study to test the hypothesis that methylation in peripheral blood cell DNA may be a predictor of breast cancer risk. Recently, two alterations in the pattern of DNA methylation in breast cancer have been described. First, active estrogen receptor-α (ER-α), which reflects breast cancer risk, is associated with decreased methylation of ER-α target genes (ERT) [12], [13]. Second, polycomb group target genes (PCGT), which play a key role in stem cell biology, are more likely to have promoter DNA hypermethylation in cancer than other genes [14], [15], [16]. These observations led to our hypothesis that the pattern of peripheral blood cell DNA methylation of ERT genes and PCGT genes is an important predictor of breast cancer risk. Based on our previous data we hypothesized that as a function of time and dose, cumulative estrogen exposure during lifetime leaves an epigenetic signature in peripheral white blood cell DNA (depending on a woman's genetic and lifestyle background), which is associated with a postmenopausal risk to develop breast cancer. Physiologically, aging and decrease of estrogens in the postmenopause may lead to methylation of ERT genes. However, lack of methylation may be an indicator for a long-term high estrogen exposure, thus the expectation would be that ERT genes have a lower frequency of methylation in peripheral blood cell DNA of women with breast cancer compared to controls. Furthermore, embryonic stem cells rely on polycomb group proteins to reversibly repress genes required for differentiation. We recently reported that stem cell polycomb group targets are up to 12-fold more likely to have cancer-specific promoter DNA hypermethylation [14], supporting a stem cell origin of cancer where reversible gene expression is replaced by permanent silencing, locking the cell into a perpetual state of self-renewal that predisposes to subsequent malignant transformation. Based on this, our expectation would be that PCGT genes have a higher frequency of methylation in peripheral blood cell DNA of women with breast cancer compared to controls.

In this study we have demonstrated that methylation in peripheral blood cell DNA is related to breast cancer risk. This observation opens opportunities for progress in risk assessment and prevention of breast cancer and is a model for investigation in other chronic diseases.

## Results and Discussion

In order to test our hypothesis that peripheral blood cell DNA methylation is predictor of breast cancer risk, we took a three step approach. In step 1 a total of 49 genes were selected as shown in Table 1. These include (a) 19 ERT genes, previously reported to be associated with decreased DNA methylation [12], [13], (b) 4 genes which are not known to be ER-α target genes, but have been demonstrated to be differently methylated depending on hormone receptor status (DMHR) [12], (c) 20 PCGT genes, which play a key role in stem cell biology and are more likely to have promoter DNA hypermethylation in cancer [14], [15], [16] and (d) 6 genes which are known to be methylated in breast cancer (MBC) [12] and are not a member of the other three groups.

**Table 1:** Gene loci selected for the case/control study.

Gene group	Gene loci	Gene ID Accession #	% methylated	Gene group	Gene loci	Gene ID Accession #	% methylated
			(*n* = 83)				(*n* = 83)
**ERT**	*BCL2*	596	0	**PCGT**	*ITGA4*	3676	0
	*MGA (II)*	23269	0		*CDKN2C*	1031	0
	*NUP155 (II)*	9631	1		*TWIST1*	7291	1
	*BRIP1 (II)*	83990	6		SLC6A20*	54716	5
	*ZC3H4 (C19orf7)*	23211	7		CYP1B1*	1545	11
	*MGA (I)*	23269	11		NEUROG1*	4762	14
	BRIP1 (I)*	83990	13		HOXA1*	3198	23
	ESR1*	2099	33		TITF1*	7080	23
	SIRT3*	23410	36		GDNF*	2668	24
	NUP155 (I)*	9631	41		NEUROD1*	4760	31
	PITX2 (II)*	5308	41		SFRP1*	6422	41
	PITX2 (I)*	5308	42		MYOD1*	4654	59
	DCC*	1630	46		*CALCA*	796	64
	ZNF217 (II)*	7764	54		*HOXA10*	3206	64
	FLJ39739*	388685	58		*SFRP4*	6424	69
	PGR*	5241	77		*ZBTB16*	7704	71
	*TRIP10*	9322	95		*SLIT2*	9353	72
	*ZNF217 (I)*	7764	99		*HIC1*	3090	82
	*TFF1*	7031	100		*HOXA11*	3207	82
**MBC**	*TERT*	7015	0		*SFRP5*	6425	83
	SYK*	6850	7				
	SEZ6L*	23544	45	**DMHR**	TIMP3*	7078	12
	*GATA5*	140628	61		CDH13*	1012	20
	*SFRP2*	6423	76		HSD17B4*	3295	27
	*CCND2*	894	82		PTGS2*	5743	77

Gene loci (either one locus or two separate loci of the same gene) have been analyzed and were selected based on their frequency of methylation in peripheral blood cell DNA in the general population of 83 healthy postmenopausal women. Genes that were selected for further analysis in the case/control study are indicated with an asterisk. ERT = estrogen receptor-α target; DMHR = differently methylated depending on hormone receptor status; PCGT = stem cell polycomb group target; MBC = methylated in breast cancer.

In step 2 we used MethyLight [17] to analyze the 49 genes (Table 1; additional information see Table S1a–b) in peripheral blood cell DNA of 83 healthy postmenopausal women (participating in UKOPS; see Materials and Methods) blinded for any history of cancer. DNA methylation at each locus (expressed as percentage of methylated reference; PMR) was divided into methylated (PMR>0) and unmethylated (PMR = 0), given that methylation was present only in a minority of women for most genes. Based on the hypothesis that lack of methylation of ERT (and DMHR) and methylation of PCGT genes predict breast cancer risk, for further analysis in the case-control study (step 3), we selected those ERT loci with a higher frequency in methylation and those PCGT loci with a lower frequency of methylation in the healthy postmenopausal women analyzed. All 4 DHMR and the 2 MBC gene loci with a lower frequency of methylation in the healthy individuals were also selected for further analysis. On this basis a total of 25 genes were further analyzed in a case-control population (indicated with an asterisk in Table 1).

Step 3 involved conducting a carefully designed case-control study involving 1,083 samples from both healthy women and breast cancer patients provided from the ESTHER study (see Material and Methods). 353 cases and 730 age-matched controls, recruited in a state-wide study in Saarland, Germany, were included in the study. Cases were more likely to have first-degree relatives with breast cancer (odds ratio (OR) 1.90 (95% confidence interval (CI) 1.27–2.85; *P* = 0.002). In addition, there was a trend towards increased breast cancer risk for women with late menopause (50+ vs. <50 yrs. OR 1.31 (95% CI 0.98–1.75; *P* = 0.10). There was no significant difference between cases and controls regarding other features (Table 2). Results were essentially unchanged when all continuous covariates were entered as such in the model. A multiple logistic regression model based on the risk factors addressed in table 2 showed a concordance statistic [18] of 0.585, which is identical with the concordance statistics demonstrated for the Gail model [2]. Jointly, the various risk factors contributed significantly to the prediction of disease status (*P*-value for likelihood ratio test = 0.007, 12 degrees of freedom). Clinicopathological features of the cases are equivalent to an average cohort of women with breast cancer (Table S2).

**Table 2:** Breast cancer risk factors in cases and controls.

Characteristics		Cases		Controls		OR***	95% CI		*P*-value
		*n*	%	*n*	%		from	to	
**Age (matching variable)**	50–54	48	13.6	100	13.7				
	55–59	65	18.4	129	17.7				
	60–64	99	28.1	211	28.9				
	65–69	88	24.9	182	24.9				
	70–74	53	15.0	108	14.8				
**1st degree relative with breast cancer**	no	302	85.6	671	91.9	1.00			
	yes	51	14.4	59	8.1	1.90	1.27	2.85	0.002**
Age at menarche*	<12	29	8.9	85	12.5	1.00			
	12–13	132	40.6	264	38.8	1.45	0.91	2.31	
	14+	164	50.5	331	48.7	1.44	0.88	2.36	0.39
Age at 1st childbirth*	<20	34	9.8	77	10.9	1.00			
	20–24	155	44.8	361	51.3	0.95	0.61	1.49	
	25–29	77	22.3	145	20.6	1.11	0.67	1.84	
	30+	80	23.1	121	17.2	1.28	0.76	2.15	0.18
Age at menopause*	<50	172	52.3	403	58.8	1.00			
	50+	157	47.7	283	41.2	1.31	0.98	1.75	0.10
BMI at age 20*	<18.5	61	17.9	101	15.1	1.00			
	18.5−<22.5	190	55.7	369	55.3	0.88	0.61	1.28	
	22.5+	90	26.4	197	29.5	0.86	0.54	1.23	0.34
Ever breastfed*	no	148	42.5	263	36.9	1.00			
	yes	200	57.5	449	63.1	0.85	0.64	1.13	0.20
Ever use of hormone replacement therapy*	no	152	46.8	348	50.4	1.00			
	yes	173	53.2	343	49.6	1.15	0.87	1.51	0.27

Cases (n = 353) and controls (n = 730) significantly differed regarding presence of 1^st^ degree relatives with breast cancer. There was also a trend towards increased risk for women with older age at first live birth and late menopause. ^*^numbers do not add up to total numbers due to missing values for the following covariates (numbers of missing values in cases/controls): age at menarche (28/50), age at 1^st^ childbirth (7/26), age at menopause (24/44), BMI at age 20 (12/63), ever breastfed (5/18), ever use of hormone replacement therapy (28/39); ^**^ indicates *P*-values <0.05; ^***^adjusted for other variables in table and taking care of missing values by multiple imputation; OR = odds ratio; CI = confidence interval, BMI body mass index.

Using PMR values, 7 of the 25 genes analyzed in stage 3 demonstrated significant differences between the 353 cases and 730 controls (Table S3). 6 of these 7 genes retained their significant differences after dividing each locus into methylated (PMR>0) or unmethylated (PMR = 0) and adjusting for age (Table 3). 5 of those were significantly associated with breast cancer risk even after adjusting for family history of breast cancer (Table 3), and after additional control for all the other established breast cancer risk factors (*ZNF217:* OR 1.48, 95% CI 1.12–1.97, *P* = 0.006; *NEUROD1:* OR 1.49, 95% CI 1.11–2.02, *P* = 0.009; *SFRP1*: OR 1.44, 95% CI 1.07–1.92, *P* = 0.015; *TITF1*: OR 1.49, 95% CI 1.02–2.18, *P* = 0.038; *NUP155:* OR 1.40, 95% CI 1.02–1.94, *P* = 0.040). A model solely based on the methylation markers of all 25 loci (methylated vs. unmethylated) showed a concordance statistic of 0.628. Jointly, the methylation status of the 25 genes was significantly related to disease risk (*P*-value for likelihood ratio test with 25 degrees of freedom = 0.048). The combination of both risk factors and methylation status increased the concordance statistic to 0.647. The likelihood ratio test for adding traditional risk factors (family history, age at menarche, age at 1^st^ childbirth, age at menopause, BMI at age 20, history of breastfeeding, history of hormone replacement therapy, 7 degrees of freedom) to a model including methylation status of the 25 genes and the matching factor age yielded a *P*-value of 0.011. Conversely, the likelihood ratio test for adding methylation status of the 25 genes (25 degrees of freedom) to a model including traditional risk factors yielded a *P*-value of 0.057.

**Table 3:** DNA methylation and odds ratios for breast cancer risk associated with lack of DNA methylation.

Gene group	Gene loci	Presence of methylation in cases (all)	Presence of methylation in controls	Adjusted for age		Adjusted for age and family history	
				OR (95% CI)	*P*-value	OR (95% CI)	*P*-value
**ERT**	*BRIP1 (I)*	31%(95/306)	30.5%(199/653)	0.97(0.72–1.31)	0.852	0.97(0.72–1.31)	0.85
	*ESR1*	12.2%(39/320)	13.5%(91/676)	1.1(0.73–1.65)	0.645	1.09(0.73–1.64)	0.678
	*SIRT3*	20.7%(61/294)	16.3%(102/627)	0.74(0.52–1.06)	0.1	0.74(0.52–1.06)	0.099
	***NUP155 (I)***	**21.9%(67/306)**	**28.6%(187/653)**	**1.42(1.03–1.96)**	0.031*	**1.4(1.02–1.94)**	0.038*
	*PITX2 (I)*	33.1%(106/320)	38%(257/676)	1.24(0.94–1.64)	0.133	1.23(0.92–1.63)	0.157
	*PITX2 (II)*	48.1%(154/320)	48.5%(328/676)	1.01(0.78–1.33)	0.916	1.02(0.78–1.33)	0.913
	*DCC*	35.1%(106/302)	41.7%(266/638)	1.32(0.99–1.75)	0.059	1.29(0.96–1.71)	0.086
	***ZNF217 (II)***	**39.4%(119/302)**	**48.9%(312/638)**	**1.49(1.13–1.97)**	0.005*	**1.49(1.12–1.97)**	0.006*
	*FLJ39739*	52%(153/294)	49.4%(310/627)	0.9(0.68–1.19)	0.474	0.9(0.68–1.19)	0.454
	*PGR*	69.7%(223/320)	70.7%(478/676)	1.05(0.78–1.4)	0.754	1.04(0.77–1.39)	0.808
**DMHR**	*TIMP3*	12.5%(40/320)	14.2%(96/676)	1.14(0.77–1.7)	0.511	1.13(0.76–1.69)	0.537
	*CDH13*	13.8%(44/320)	15.4%(104/676)	1.15(0.78–1.68)	0.485	1.15(0.79–1.69)	0.462
	*HSD17B4*	17.5%(56/320)	14.3%(97/676)	0.79(0.55–1.13)	0.19	0.78(0.55–1.13)	0.189
	***PTGS2***	**71.3%(228/320)**	**77.1%(521/676)**	**1.35(1–1.83)**	0.049*	1.31(0.97–1.78)	0.078
**PCGT**	*SLC6A20*	0.9%(3/320)	1.8%(12/676)	1.94(0.54–6.94)	0.307	2.2(0.61–7.96)	0.23
	*NEUROG1*	4.3%(13/302)	4.7%(30/638)	1.1(0.56–2.14)	0.783	1.08(0.55–2.11)	0.822
	*HOXA1*	11.1%(34/306)	13.9%(91/653)	1.28(0.84–1.96)	0.247	1.27(0.83–1.94)	0.272
	***TITF1***	**13.8%(44/320)**	**19.5%(132/676)**	**1.53(1.05–2.22)**	0.025*	**1.52(1.05–2.21)**	0.028*
	*GDNF*	14.7%(45/306)	18.7%(122/653)	1.32(0.91–1.92)	0.147	1.3(0.89–1.89)	0.176
	***NEUROD1***	**30.5%(91/298)**	**38.9%(250/642)**	**1.45(1.08–1.94)**	0.014*	**1.47(1.09–1.98)**	0.011*
	***SFRP1***	**29.4%(94/320)**	**37.4%(253/676)**	**1.44(1.08–1.91)**	0.014*	**1.44(1.08–1.93)**	0.013*
	*MYOD1*	60%(192/320)	63.5%(429/676)	1.18(0.89–1.55)	0.244	1.17(0.89–1.54)	0.261
**MBC**	*SYK*	2.2%(7/320)	2.4%(16/676)	1.07(0.43–2.62)	0.889	1.06(0.43–2.62)	0.9
	*CYP1B1*	7.5%(24/320)	4.9%(33/676)	0.63(0.37–1.09)	0.096	0.62(0.36–1.08)	0.09
	*SEZ6L*	50.3%(154/306)	52.8%(345/653)	1.1(0.84–1.45)	0.482	1.1(0.83–1.44)	0.517

6 genes analyzed showed differences in the methylation pattern between cases and controls when the data was adjusted for age; 5 remained significant even after adjusting for age and family history of breast cancer. ERT = estrogen receptor-α target; DMHR = differently methylated depending on hormone receptor status; PCGT = stem cell polycomb group target; MBC = methylated in breast cancer; ^*^ indicates *P* values <0.05.

Taken together, these patterns suggest exactly what we had expected based on the selection of genes we used for this study: The prediction of risk by traditional risk factors and methylation patterns are not entirely independent. This again supports our hypothesis that DNA methylation, on one hand may act as a surrogate for genetic risk and lifelong environmental exposure, but on the other hand, independently reflects the individual response of women to these factors. In this context the results of the first genome-wide-breast cancer susceptibility study which was recently reported are relevant. The study identified 5 novel breast cancer susceptibility loci after a three stage procedure, which started with analysis of 227,876 single nucleotide polymorphisms (SNPs). The per allele odds ratio for an association with breast cancer for the top five SNPs was between 1.07 and 1.26 [19]. This study suggests that it is unlikely that genetic tests alone will achieve the sensitivity and specificity required for risk assessment to guide preventive measures.

Furthermore, the two main histological subtypes in breast cancer are invasive ductal and invasive lobular. It is known that there are specific carcinogenic pathways for these two breast cancer groups. There is growing evidence suggesting that a change in tumor tissue architecture takes place in the genesis of invasive lobular breast cancer known as epithelial-mesenchymal transition [20]. To study whether this is also reflected in the epigenotype of peripheral blood cells, we calculated separate ORs which predict each histological subtype (Table S4a–b). The genes which predicted invasive ductal breast cancer (*ZNF217*, *NEUROD1*, *SFRP1*) were completely different from the genes associated with invasive lobular cancer (*PITX2*, *TITF1*, *GDNF*, *MYOD1*, *DCC*). Interestingly genes which predicted invasive lobular cancer are involved in diseases that affect the mesenchyme like Hirschsprung disease or are known to be involved in epithelial-mesenchymal transition [21]–[25].

Beside histology, estrogen receptor status is the most important feature in breast cancer. It is known that estrogen exposure (e.g. serum estradiol in postmenopausal women) is only associated with ER positive breast cancer [26]. Therefore we tested which of the 25 genes predict ER positive and which predict ER negative breast cancer. Interestingly only the ERT gene loci (*ZNF217* OR 1.48 (95% CI 1.01–2.16; *P* = 0.042) and *NUP155* OR 1.46 (95% CI 1.05–2.02; *P* = 0.023) predicted ER positive breast cancer (Table 4), whereas a PCGT gene locus (*SFRP1* OR 2.37 (95% CI 1.23–4.57; *P* = 0.01) predicted ER negative breast cancer (Table 5).

**Table 4:** Odds ratios for estrogen receptor positive breast cancer risk associated with lack of DNA methylation.

Gene group	Gene loci	Presence of methylation in cases (ER positive)	Presence of methylation in controls	Adjusted for age		Adjusted for age and family history	
				OR (95% CI)	*P*- value	OR (95% CI)	*P*- value
**ERT**	*BRIP1 (I)*	30% (61/203)	30.5% (199/653)	1.01 (0.72–1.43)	0.943	1.01 (0.72–1.43)	0.942
	*ESR1*	11.3% (24/212)	13.5% (91/676)	1.23 (0.76–1.99)	0.404	1.22 (0.75–1.98)	0.419
	*SIRT3*	18.6% (36/194)	16.3% (102/627)	0.84 (0.55–1.27)	0.405	0.83 (0.54–1.27)	0.384
	***NUP155 (I)***	**21.2% (43/203)**	**28.6% (187/653)**	**1.48 (1.01–2.16)**	0.042*	**1.46 (1–2.13)**	**0.052**
	*PITX2 (I)*	32.5% (69/212)	38% (257/676)	1.27 (0.92–1.77)	0.151	1.25 (0.9–1.74)	0.189
	*PITX2 (II)*	49.1% (104/212)	48.5% (328/676)	0.99 (0.73–1.35)	0.951	0.97 (0.71–1.33)	0.865
	*DCC*	37.5% (75/200)	41.7% (266/638)	1.19 (0.86–1.66)	0.291	1.15 (0.82–1.6)	0.41
	***ZNF217 (II)***	**40% (80/200)**	**48.9% (312/638)**	**1.46 (1.05–2.02)**	0.023*	**1.44 (1.04–1.99)**	0.029*
	*FLJ39739*	52.6% (102/194)	49.4% (310/627)	0.88 (0.63–1.21)	0.43	0.87 (0.63–1.21)	0.413
	*PGR*	69.3% (147/212)	70.7% (478/676)	1.07 (0.76–1.5)	0.69	1.05 (0.75–1.47)	0.789
**DMHR**	*TIMP3*	11.3% (24/212)	14.2% (96/676)	1.27 (0.79–2.05)	0.327	1.26 (0.78–2.04)	0.347
	*CDH13*	12.7% (27/212)	15.4% (104/676)	1.28 (0.81–2.01)	0.296	1.28 (0.81–2.03)	0.288
	*HSD17B4*	17% (36/212)	14.3% (97/676)	0.81 (0.53–1.23)	0.323	0.8 (0.53–1.23)	0.31
	*PTGS2*	72.2% (153/212)	77.1% (521/676)	1.31 (0.92–1.87)	0.129	1.26 (0.88–1.79)	0.205
**PCGT**	*SLC6A20*	0.9% (2/212)	1.8% (12/676)	1.98 (0.44–8.92)	0.376	2.31 (0.5–10.61)	0.28
	*NEUROG1*	5% (10/200)	4.7% (30/638)	0.91 (0.43–1.9)	0.794	0.89 (0.43–1.87)	0.762
	*HOXA1*	11.3% (23/203)	13.9% (91/653)	1.26 (0.77–2.06)	0.349	1.25 (0.76–2.04)	0.378
	*TITF1*	15.6% (33/212)	19.5% (132/676)	1.34 (0.88–2.04)	0.173	1.33 (0.87–2.02)	0.189
	*GDNF*	15.8% (32/203)	18.7% (122/653)	1.23 (0.8–1.89)	0.339	1.2 (0.78–1.85)	0.406
	*NEUROD1*	32% (63/197)	38.9% (250/642)	1.35 (0.96–1.9)	0.085	1.37 (0.97–1.94)	0.071
	*SFRP1*	32.1% (68/212)	37.4% (253/676)	1.26 (0.91–1.76)	0.165	1.27 (0.91–1.77)	0.161
	*MYOD1*	59% (125/212)	63.5% (429/676)	1.24 (0.91–1.71)	0.177	1.23 (0.89–1.69)	0.203
**MBC**	*SYK*	2.8% (6/212)	2.4% (16/676)	0.82 (0.32–2.14)	0.691	0.82 (0.31–2.15)	0.688
	*CYP1B1*	8% (17/212)	4.9% (33/676)	0.58 (0.31–1.06)	0.075	0.57 (0.31–1.06)	0.076
	*SEZ6L*	50.2% (102/203)	52.8% (345/653)	1.11 (0.81–1.53)	0.514	1.1 (0.8–1.51)	0.569

Only the estrogen receptor regulated genes (*NUP155* and *ZNF217*) predicted the estrogen receptor positive breast cancer, which fits with the general hypothesis that estrogen exposure is associated to breast cancer risk. The data is adjusted for age alone or for age and family history of breast cancer.ERT = estrogen receptor-α target; DMHR = differently methylated depending on hormone receptor status; PCGT = stem cell polycomb group target; MBC = methylated in breast cancer; ER = estrogen receptor; OR = odds ratio; CI = confidence interval; ^*^ indicates *P*-values <0.05.

**Table 5:** Odds ratios for estrogen receptor negative breast cancer risk associated with lack of DNA methylation.

Gene group	Gene loci	Presence of methylation in cases (ER negative)	Presence of methylation in controls	Adjusted for age		Adjusted for age and family history	
				OR (95% CI)	*P*-value	OR (95% CI)	*P*- value
**ERT**	*BRIP1 (I)*	31.6% (18/57)	30.5% (199/653)	0.95 (0.53–1.72)	0.875	0.95 (0.53–1.71)	0.866
	*ESR1*	11.7% (7/60)	13.5% (91/676)	1.07 (0.47–2.43)	0.88	1.06 (0.47–2.43)	0.881
	*SIRT3*	24.6% (14/57)	16.3% (102/627)	0.64 (0.34–1.22)	0.174	0.64 (0.34–1.23)	0.18
	*NUP155 (I)*	21.1% (12/57)	28.6% (187/653)	1.51 (0.78–2.92)	0.224	1.51 (0.78–2.92)	0.225
	*PITX2 (I)*	33.3% (20/60)	38% (257/676)	1.24 (0.7–2.18)	0.457	1.23 (0.7–2.17)	0.468
	*PITX2 (II)*	46.7% (28/60)	48.5% (328/676)	1.04 (0.61–1.78)	0.873	1.04 (0.61–1.78)	0.876
	*DCC*	29.8% (17/57)	41.7% (266/638)	1.67 (0.92–3.01)	0.091	1.66 (0.91–3)	0.096
	*ZNF217 (II)*	36.8% (21/57)	48.9% (312/638)	1.59 (0.91–2.8)	0.106	1.61 (0.91–2.83)	0.1
	*FLJ39739*	49.1% (28/57)	49.4% (310/627)	1.02 (0.59–1.77)	0.934	1.03 (0.59–1.78)	0.926
	*PGR*	73.3% (44/60)	70.7% (478/676)	0.9 (0.49–1.64)	0.735	0.9 (0.49–1.64)	0.732
**DMHR**	*TIMP3*	16.7% (10/60)	14.2% (96/676)	0.87 (0.42–1.79)	0.708	0.87 (0.42–1.79)	0.706
	*CDH13*	15% (9/60)	15.4% (104/676)	1.03 (0.49–2.17)	0.933	1.03 (0.49–2.16)	0.941
	*HSD17B4*	21.7% (13/60)	14.3% (97/676)	0.59 (0.31–1.14)	0.119	0.59 (0.31–1.14)	0.119
	*PTGS2*	71.7% (43/60)	77.1% (521/676)	1.3 (0.72–2.36)	0.38	1.3 (0.72–2.35)	0.387
**PCGT**	*SLC6A20*	0% (0/60)	1.8% (12/676)				
	*NEUROG1*	0% (0/57)	4.7% (30/638)				
	*HOXA1*	14% (8/57)	13.9% (91/653)	0.97 (0.44–2.14)	0.947	0.97 (0.44–2.12)	0.932
	*TITF1*	8.3% (5/60)	19.5% (132/676)	2.53 (0.99–6.48)	0.053	2.53 (0.99–6.48)	0.053
	*GDNF*	10.5% (6/57)	18.7% (122/653)	1.85 (0.77–4.43)	0.168	1.84 (0.77–4.4)	0.172
	*NEUROD1*	28.1% (16/57)	38.9% (250/642)	1.66 (0.91–3.04)	0.098	1.66 (0.91–3.04)	0.099
	***SFRP1***	**20% (12/60)**	**37.4% (253/676)**	**2.37 (1.23–4.57)**	0.01*	**2.37 (1.23–4.57)**	0.01*
	*MYOD1*	65% (39/60)	63.5% (429/676)	0.94 (0.54–1.65)	0.839	0.94 (0.54–1.65)	0.828
**MBC**	*SYK*	1.7% (1/60)	2.4% (16/676)	1.28 (0.16–9.91)	0.816	1.26 (0.16–9.79)	0.826
	*CYP1B1*	6.7% (4/60)	4.9% (33/676)	0.71 (0.24–2.09)	0.529	0.71 (0.24–2.1)	0.534
	*SEZ6L*	50.9% (29/57)	52.8% (345/653)	1.07 (0.62–1.85)	0.799	1.07 (0.62–1.84)	0.815

*SFRP1*, a polycomb group target gene unrelated to the hormonal exposure (estrogen), predicted the estrogen receptor negative breast cancer status. The data was significant after adjusting for age alone or for age and family history of breast cancer. ERT = estrogen receptor-α target; DMHR = differently methylated depending on hormone receptor status; PCGT = stem cell polycomb group target; MBC = methylated in breast cancer; ER = estrogen receptor; OR = odds ratio; CI = confidence interval; ^*^ indicates *P*-values <0.05.

**Figure 1:**
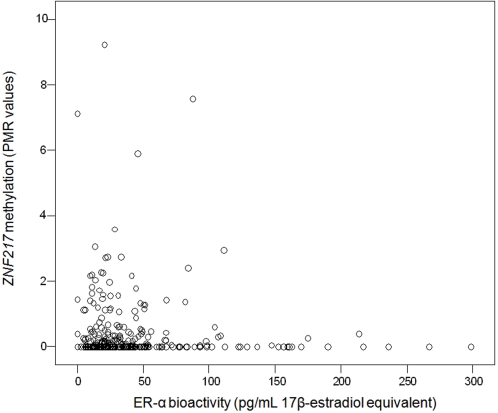
Association of ER-α bioactivity of serum with DNA methylation of *ZNF217* in the corresponding peripheral blood cell DNA. Peripheral blood cell DNA methylation of *ZNF217*, one of the estrogen receptor target genes studied, demonstrated a significant inverse correlation with ER-α bioactivity in the corresponding serum (*ρ* = −0.112; *P* = 0.046).

Given the relationship between ERT genes and breast cancer risk we considered the possibility that current serum estrogen activity may have an impact on DNA methylation of these genes. To assess this possibility we analyzed ER-α bioactivity in the serum of the cases and controls using a functional assay, which measures the potential for binding to ER-α and transactivating the estrogen responsive elements (EREs). This ER-α ERE-GFP (green fluorescent protein) reporter test system in *Saccharomyces cerevisiae* has been recently described [27], [28]. Only peripheral blood cell DNA methylation of *ZNF217*, one of the 10 ERT genes, demonstrated a significant inverse correlation (*ρ* = −0.112; *P* = 0.046) with ER-α bioactivity in the corresponding serum (Figure 1). The possibility that this correlation could be triggered by the current use of hormone replacement therapy was considered. After excluding women currently taking hormone replacement therapy, the inverse correlation remained significant (*ρ* = −0.166; *P* = 0.013). Our data suggest that lack of *ZNF217* methylation is a long-term surrogate marker of estrogen exposure, indicating a woman's risk of breast cancer. This observation fits with evidence for changes in the activity of ZNF217 in breast carcinogenesis. *ZNF217* encodes a transcription factor which mainly represses genes involved in differentiation [29]. Introduction of ZNF217 into human mammary epithelial cells leads to immortalization [30]. In addition, *ZNF217* is amplified and overexpressed in 40% and 18% of breast cancer cell lines and tissues, respectively [31], [32].

Interestingly, analyzing solely women currently taking hormone replacement therapy, *PGR*, which is also an ERT gene, was the only locus demonstrating significant inverse correlation with corresponding serum ER-α bioactivity (*ρ* = −0.242; *P* = 0.044). Peripheral blood cell DNA methylation of *PGR*, the gene coding for the progesterone receptor whose expression is strongly regulated by ER-α, was not linked to breast cancer risk. *PGR* methylation is not significantly different between non-neoplastic breast tissue and breast cancer in premenopausal women [14], but *PGR* methylation analyzed in breast cancer tissue adjusted for age and expression of progesterone receptor is the most significant predictor of ER status in breast cancer [12]. Using logistic regression and after adjustment for age and progesterone receptor status, lack of peripheral blood cell *PGR* methylation predicted the ER status of the corresponding breast cancer (OR 1.52; 95% CI 1.10–2.11; *P* = 0.012), whereas none of the other 24 gene loci's methylation status was an ER status predictor. This finding indicates that the epigenetic pathways and interactions, which have previously only been identified in cancer tissue, are also reflected in peripheral blood, a source of DNA completely unrelated to the breast cancer tissue.

The results did not however confirm the hypothesis that peripheral blood cell DNA methylation of PCGT genes is associated with an increased breast cancer risk. One possible explanation for this could be that lack of methylation at specific gene loci is only a surrogate for general global hypomethylation. This hypothesis is supported by data demonstrating that lack of folic acid supply (a condition leading to depletion of available methyl groups and subsequent global hypomethylation) is associated with some forms of cancer [33]. In order to study global methylation we analyzed peripheral blood cell DNA methylation of ALU repetitive elements, which has recently been demonstrated to be an excellent marker for global methylation [34]. None of the single predictive loci was associated with global methylation in the 349 samples analyzed for methylation of ALU (Table 6). ALU methylation did not differ between cases and controls (controls (*n* = 180), median PMR 54.8 and interquartile range 34.0–84.0; cases (*n* = 169), median PMR 51.1 and interquartile range 34.7–73.0; Wilcoxon-Mann-Whitney test *P* = 0.25). This again indicates that the predictive potential of DNA methylation is only reflected by specific gene loci rather than by a surrogate marker which reflects global single-carbon metabolism.

**Table 6:** Correlation of global methylation (ALU repetitive elements) with methylation at single gene loci.

Gene group	Gene loci	Cases & Controls		Cases only		Controls only	
		*n* = 349		*n* = 169		*n* = 180	
		rho	*P*-value	rho	*P*-value	rho	*P*-value
**ERT**	*BRIP1 (I)*	0.037	0.501	0.035	0.657	0.047	0.543
	***ESR1***	**0.125**	0.0204*	0.162	0.0361*	0.105	0.164
	*SIRT3*	0.062	0.272	0.001	0.987	0.115	0.142
	*NUP155 (I)*	0.004	0.946	0.031	0.692	−0.026	0.732
	*PITX2 (I)*	0.017	0.752	0.031	0.691	0.006	0.938
	*PITX2 (II)*	0.019	0.720	−0.087	0.263	0.120	0.111
	*DCC*	0.040	0.477	0.026	0.740	0.054	0.493
	*ZNF217 (II)*	0.023	0.681	−0.012	0.880	0.041	0.601
	*FLJ39739*	−0.048	0.395	−0.080	0.319	−0.012	0.875
	*PGR*	−0.061	0.255	−0.149	0.055	0.014	0.851
**DMHR**	*TIMP3*	0.028	0.780	−0.023	0.772	0.058	0.441
	*CDH13*	−0.060	0.267	0.045	0.560	−0.146	0.052
	*HSD17B4*	0.012	0.828	0.115	0.138	−0.095	0.206
	*PTGS2*	0.094	0.082	0.119	0.126	0.070	0.356
**PCGT**	*SLC6A20*	−0.030	0.578	−0.063	0.416	−0.006	0.937
	*NEUROG1*	0.062	0.261	0.034	0.692	0.109	0.162
	*HOXA1*	−0.049	0.373	0.042	0.598	−0.131	0.087
	*TITF1*	−0.015	0.780	−0.019	0.804	−0.011	0.884
	*GDNF*	−0.029	0.599	−0.051	0.518	−0.020	0.799
	*NEUROD1*	0.037	0.509	0.064	0.423	0.001	0.986
	*SFRP1*	−0.046	0.396	−0.148	0.056	0.045	0.553
	*MYOD1*	0.043	0.431	−0.026	0.742	0.099	0.189
**MBC**	*SYK*	0.007	0.901	0.057	0.465	−0.031	0.682
	*CYP1B1*	0.074	0.267	0.080	0.306	0.068	0.364
	***SEZ6L***	**−0.129**	0.018*	−0.167	0.034*	−0.093	0.227

Analysis of ALU methylation in a subset of cases (n = 169) and controls (n = 180) demonstrated no significant association with the predictive loci *(NUP155*, *ZNF217*, *PTGS2*, *TITF1*, *NEUROD1*, *SFRP1)*, supporting the role of DNA methylation at particular loci as a valuable predictive marker for breast cancer risk.ERT = estrogen receptor-α target; DMHR = differently methylated depending on hormone receptor status; PCGT = stem cell polycomb group target; MBC = methylated in breast cancer; ^*^ indicates *P*-values <0.05.

It is worth noting that the samples used in this study were obtained after the diagnosis of breast cancer. In women with metastatic breast cancer, tumor cells (5 tumor cells in 7.5 mL blood) can be identified in the peripheral blood [35]. This means that on average 1 out of 40 million cells in the peripheral blood of women with metastatic (less in non-metastatic) are breast cancer cells. Although MethyLight is very sensitive, this assay is only capable of detecting methylated alleles in the presence of a 10,000 -fold excess of unmethylated alleles [17]. The possibility that the methylation signal we detect in peripheral blood cell DNA is influenced by tumor cell DNA can therefore be discounted.

In this first large scale epigenotyping study, we were able to demonstrate that particular DNA methylation patterns in peripheral blood may serve as a surrogate marker for breast cancer risk. The current report also provides a basis for further research to assess the role of a combination of genotyping and epigenotyping as a clinical tool to predict an individual's risk of developing breast cancer, other cancers and chronic diseases with sufficient accuracy to guide preventive measures.

## Materials and Methods

### Samples

**UKOPS** (United Kingdom Ovarian Cancer Population Study) study is being carried out in 10 regional centers in England, Wales and Northern Ireland. Recruitment has started since January 2006 and is aiming to include 2,000 ovarian cancer patients, 1,500 benign and 5,000 controls. Recruitment of cancer cases has been carried out during visits to the Gynecological Oncology wards. Control participants have been recruited from the UKCTOCS (United Kingdom Collaborative Trial of Ovarian Cancer Screening) trial centers (www.ukctocs.org.uk). Detailed information about the medical history was obtained by a standardized questionnaire. Diagnosis data from histology and cytology reports has also been included. Furthermore, serum and whole blood were collected from all subjects. Written consent was obtained from each participant. Ethical approval was received by the joint University College London Committees on the Ethics of Human Research (Committee A). In the current study 83 healthy postmenopausal women (blinded for cancer history) were selected for step 2.

**ESTHER** (www.esther.dkfz.org/esther/) is a population-based study carried out in the state of Saarland (located in South West Germany). In the clinical arm of the study, 1,981 cancer patients age 50 to 75, including 380 women with breast cancer, were recruited during their first stay in the hospital for primary cancer treatment. In the community arm, of the study, 9,953 women and men age 50–75 were recruited during routine health examinations by their general practitioners. Recruitment and baseline examinations were carried out in 2000–2003. In both study arms, detailed information about medical history, including family history, sociodemographic and lifestyle factors and current health status was obtained by standardized questionnaire. In addition, serum and whole blood samples were collected. Written informed consent was obtained from all subjects. The protocol was approved by the Ethics Committee of the Medical Faculty of the University of Heidelberg. Germany. In this study, all 353 cases with postmenopausal invasive breast cancer were included, and a stratified random subset of 730 age-matched postmenopausal women were selected as controls.

### DNA isolation and storage of DNA

A standard chloroform-based DNA isolation protocol has been used to extract DNA from whole blood from the UKOPS samples. DNA was extracted from whole blood samples of the ESTHER study participants by Invisorb extraction kits (Invitek; www.invitek.de). DNA from both sample collections have been dissolved in distilled water and stored at −80°C until analysis.

### Analysis of DNA Methylation

Sodium bisulfite conversion (Zymo Research; www.zymoresearch.com), MethyLight analysis (Applied Biosystems; www.appliedbiosystems.com) and nucleotide sequences for most MethyLight primers and probes in the promoter or 5′ end region was described recently [12], [14], [34], [36]. Each MethyLight reaction at a specific locus covers on average 5–7 CpG dinucleotides. A detailed list of primer and probes (Metabion; www.metabion.com) for all analyzed loci is provided in Table S1. Briefly two sets of primers and probes, designed specifically for bisulfite-converted DNA, have been used: a methylated set for the gene of interest and a reference set (*COL2A1*) to normalize for input DNA. Specificity of the reactions for methylated DNA was confirmed separately using SssI (New England Biolabs; www.newenglandbiolabs.co.uk) treated human white blood cell DNA (heavily methylated). The percentage of fully methylated molecules at a specific locus were calculated by dividing the *GENE*∶*COL2A1* ratio of a sample by the *GENE*∶*COL2A1* ratio of the SssI-treated human white blood cell DNA and multiplied by 100. The abbreviation PMR (*P*ercentage of *M*ethylated *R*eference) indicates this measurement.

The analysis was performed blinded and cases and controls were randomly mixed for bisulfite treatment and real-time PCR. The concentration of bisulfite modified DNA (assessed by the level of the reference gene *COL2A1*) was the same between cases and controls.

### ER-α serum bioactivity assay

ER-α bioactivity has been as recently described [27]. Each serum has been tested blindly in quadruplicates (20 µL for each reaction in a total of 100 µL) and a mean value has been calculated based on four independent measures (done on two different days).

### Statistical Analysis

Descriptive analyses were performed on age and known risk factors for breast cancer among cases and controls and on clinical features among breast cancer cases. Percent methylated, mean and median values of PMR were calculated for the selected gene loci. Differences in PMR values between cases and controls were analyzed by means of Wilcoxon-Mann-Whitney test. In addition, odds ratios for breast cancer (overall and according to estrogen receptor status) associated with absence of methylation, adjusted for age (matching factor) and for positive family history (at least one first degree relative with breast cancer) as well as for other established breast cancer risk factors were calculated by multiple logistic regression. Multiple imputation was employed to deal with missing covariate data in multivariable analyses. Initially, a linear term for percent methylation in addition to the dichotomous term for absence of methylation was included in the models to address a potential dose-response relationship. As this term did not significantly improve prediction for any of the 25 genes assessed, it was dropped from the final models. Joint contribution of methylation status of all 25 genes to the prediction of breast cancer risk was evaluated by concordance statistics [18] and likelihood ratio tests. Spearman's rho was calculated to assess the correlation of global methylation (ALU repetitive elements) with methylation at single gene loci. All statistical analyses were done using SAS Software, version 8.2.

## Supporting Information

Table S1 Information on genes analyzed. S1A: Primers and probe sequences for MethyLight. S1B: General gene information. Gene alternative names, chromosomal location and amplicons' position relative to the transcription start site are indicated.
(0.14 MB DOC)

Table S2 Clinicopathological characteristics of breast cancer cases used for the study. UICC = Union internationale contre le cancer; *Among those with information; n/a = not applicable.
(0.05 MB DOC)

Table S3 Mean and median percentage of methylated reference (PMR) value of the 25 different loci. 7 genes of those analyzed demonstrated significant differences in the level of methylation between cases and controls, based on the quantitative results on peripheral blood cell DNA. ERT = estrogen receptor-α target; DMHR = differently methylated depending on hormone receptor status; PCGT = stem cell polycomb group target; MBC = methylated in breast cancer; * indicates P-values <0.05
(0.08 MB DOC)

Table S4 Odds ratios for invasive ductal and lobular breast cancer risk associated with peripheral blood DNA methylation. S4A: Risk for invasive ductal breast cancer. S4B: Risk for invasive lobular breast cancer.
(0.14 MB DOC)

## References

[1] Parkin DM, Bray F, Ferlay J, Pisani P (2005). Global cancer statistics.. CA Cancer J Clin.

[2] Decarli A, Calza S, Masala G, Specchia C, Palli D (2006). Gail model for prediction of absolute risk of invasive breast cancer: independent evaluation in the Florence-European Prospective Investigation Into Cancer and Nutrition cohort.. J Natl Cancer Inst.

[3] Feinberg AP, Ohlsson R, Henikoff S (2006). The epigenetic progenitor origin of human cancer.. Nat Rev Genet.

[4] Gosden RG, Feinberg AP (2007). Genetics and epigenetics–nature's pen-and-pencil set.. N Engl J Med.

[5] Jones PA, Baylin SB (2007). The epigenomics of cancer.. Cell.

[6] Widschwendter M, Jones PA (2002). DNA methylation and breast carcinogenesis.. Oncogene.

[7] Jirtle RL, Skinner MK (2007). Environmental epigenomics and disease susceptibility.. Nat Rev Genet.

[8] Anway MD, Cupp AS, Uzumcu M, Skinner MK (2005). Epigenetic transgenerational actions of endocrine disruptors and male fertility.. Science.

[9] Cui H, Cruz-Correa M, Giardiello FM, Hutcheon DF, Kafonek DR (2003). Loss of IGF2 imprinting: a potential marker of colorectal cancer risk.. Science.

[10] Hitchins MP, Wong JJ, Suthers G, Suter CM, Martin DI (2007). Inheritance of a cancer-associated MLH1 germ-line epimutation.. N Engl J Med.

[11] Suter CM, Martin DI, Ward RL (2004). Germline epimutation of MLH1 in individuals with multiple cancers.. Nat Genet.

[12] Widschwendter M, Siegmund KD, Muller HM, Fiegl H, Marth C (2004). Association of breast cancer DNA methylation profiles with hormone receptor status and response to tamoxifen.. Cancer Res.

[13] Leu YW, Yan PS, Fan M, Jin VX, Liu JC (2004). Loss of estrogen receptor signaling triggers epigenetic silencing of downstream targets in breast cancer.. Cancer Res.

[14] Widschwendter M, Fiegl H, Egle D, Mueller-Holzner E, Spizzo G (2007). Epigenetic stem cell signature in cancer.. Nat Genet.

[15] Ohm JE, McGarvey KM, Yu X, Cheng L, Schuebel KE (2007). A stem cell-like chromatin pattern may predispose tumor suppressor genes to DNA hypermethylation and heritable silencing.. Nat Genet.

[16] Schlesinger Y, Straussman R, Keshet I, Farkash S, Hecht M (2007). Polycomb-mediated methylation on Lys27 of histone H3 pre-marks genes for de novo methylation in cancer.. Nat Genet.

[17] Eads CA, Danenberg KD, Kawakami K, Saltz LB, Blake C (2000). MethyLight: a high-throughput assay to measure DNA methylation.. Nucleic Acids Res.

[18] Gail MH, Pfeiffer RM (2005). On criteria for evaluating models of absolute risk.. Biostatistics.

[19] Easton DF, Pooley KA, Dunning AM, Pharoah PD, Thompson D (2007). Genome-wide association study identifies novel breast cancer susceptibility loci.. Nature.

[20] Yang J, Mani SA, Donaher JD, Ramaswamy S, Itzykso RA (2004). Twist, a master regualator of morphogenesis, plays an essential role in tumor metastasis.. Cell.

[21] Angrist M, Bolk S, Halushka M, Lapchak PA, Chakravarti A (1996). Germline mutations in glial cell line-derived neurotrophic factor (GDNF) and RET in a Hirschsprung disease patient.. Nat Genet.

[22] Shakya R, Jho EH, Kotka P, Wu Z, Kholodilov N (2005). The role of GDNF in patterning the excretory system.. Dev Biol.

[23] Barberi T, Willis LM, Socci ND, Studer L (2005). Derivation of multipotent mesenchymal precursors from human embryonic stem cells.. PLoS Med.

[24] Garcia-Barcelo MM, Lau DK, Ngan ES, Leon TY, Liu TT (2007). Evaluation of the thyroid transcription factor-1 gene (TITF1) as a Hirschsprung's disease locus.. Ann Hum Genet.

[25] De Langhe SP, Carraro G, Tefft D, Li C, Xu X (2008). Formation and differentiation of multiple mesenchymal lineages during lung development is regulated by beta-catenin signaling.. PLoS ONE.

[26] Missmer SA, Eliassen AH, Barbieri RL, Hankinson SE (2004). Endogenous estrogen, androgen, and progesterone concentrations and breast cancer risk among postmenopausal women.. J Natl Cancer Inst.

[27] Hasenbrink G, Sievernich A, Wildt L, Ludwig J, Lichtenberg-Frate H (2006). Estrogenic effects of natural and synthetic compounds including tibolone assessed in Saccharomyces cerevisiae expressing the human estrogen alpha and beta receptors.. FASEB J.

[28] Sievernich A, Wildt L, Lichtenberg-Frate H (2004). In vitro bioactivity of 17alpha-estradiol.. J Steroid Biochem Mol Biol.

[29] Krig SR, Jin VX, Bieda MC, O'Geen H, Yaswen P (2007). Identification of genes directly regulated by the oncogene ZNF217 using chromatin immunoprecipitation (ChIP)-chip assays.. J Biol Chem.

[30] Nonet GH, Stampfer MR, Chin K, Gray JW, Collins CC (2001). The ZNF217 gene amplified in breast cancers promotes immortalization of human mammary epithelial cells.. Cancer Res.

[31] Collins C, Rommens JM, Kowbel D, Godfrey T, Tanner M (1998). Positional cloning of ZNF217 and NABC1: genes amplified at 20q13.2 and overexpressed in breast carcinoma.. Proc Natl Acad Sci USA.

[32] Kallioniemi A, Kallioniemi OP, Piper J, Tanner M, Stokke T (1994). Detection and mapping of amplified DNA sequences in breast cancer by comparative genomic hybridization.. Proc Natl Acad Sci USA.

[33] Terry P, Jain M, Miller AB, Howe GR, Rohan TE (2002). Dietary intake of folic acid and colorectal cancer risk in a cohort of women.. Int J Cancer.

[34] Weisenberger DJ, Campan M, Long TI, Kim M, Woods C (2005). Analysis of repetitive element DNA methylation by MethyLight.. Nucleic Acids Res.

[35] Cristofanilli M, Budd GT, Ellis MJ, Stopeck A, Matera J (2004). Circulating tumor cells, disease progression, and survival in metastatic breast cancer.. N Engl J Med.

[36] Weisenberger DJ, Siegmund KD, Campan M, Young J, Long TI (2006). CpG island methylator phenotype underlies sporadic microsatellite instability and is tightly associated with BRAF mutation in colorectal cancer.. Nat Genet.

