# Supplementary material for: Epigenotyping in Peripheral Blood Cell DNA and Breast Cancer Risk: A Proof of Principle Study
Source: PLoS One. 2008 Jul 16;3(7):e2656. doi: 10.1371/journal.pone.0002656 (PMC2442168; doi:10.1371/journal.pone.0002656)
Supplement: Table S1 — Information on genes analyzed. S1A: Primers and probe sequences for MethyLight. S1B: General gene information. Gene alternative names, chromosomal location and amplicons' position relative to the transcription start site are indicated. (0.14 MB DOC) [file pone.0002656.s001.doc]

**Table S1:**

**Table S1A: Primers and probes for MethyLight.**

| **HUGO Gene Nomenclature** | **Forward Primer Sequence** | **Reverse Primer Sequence** | **Probe Oligo Sequence** |
| --- | --- | --- | --- |
| ***BCL2*** | TCGTATTTCGGGATTCGGTC | AACTAAACGCAAACCCCGC | 6FAM-ACGACGCCGAAAACAACCGAAATCTACA-BHQ-1 |
| ***BRIP1 (I)*** | CCATAAACCCGCAAACTATACAAA | CGGAGTTTAGAGCGTTGTTTCG | 6FAM-TTTCCGTAAACTTCCCTCCGACTTACCTATCTAAAAA-BHQ-1 |
| ***BRIP1 (II)*** | CGCCCAATAACCCAACGA | TTTTTATTGGATGTCGAAGTTTTCG | 6FAM-ACCCGCCAAAACCAAAAATCAACCAAT-BHQ-1 |
| ***ZC3H4*** | CGCCCTAAATACCCCGACTAC | TGGTTCGGGTCGAGTCGT | 6FAM-CCCGCCGCAATCAAAAATTAACACAAAAT-BHQ-1 |
| ***CALCA*** | GTTTTGGAAGTATGAGGGTGACG | TTCCCGCCGCTATAAATCG | 6FAM-ATTCCGCCAATACACAACAACCAATAAACG-BHQ-1 |
| ***CCND2*** | GGAGGGTCGGCGAGGAT | TCCTTTCCCCGAAAACATAAAA | 6FAM-CACGCTCGATCCTTCGCCCG-BHQ-1 |
| ***CDH13*** | AATTTCGTTCGTTTTGTGCGT | CTACCCGTACCGAACGATCC | 6FAM-AACGCAAAACGCGCCCGACA-BHQ-1 |
| ***CDKN2C*** | AAATTACAACGCCGCGAAAA | CGTGCGAGATTGCGAGC | 6FAM-AAACCGAACGCCGCCCACG-BHQ-1 |
| ***CYP1B1*** | GTGCGTTTGGACGGGAGTT | AACGCGACCTAACAAAACGAA | 6FAM-CGCCGCACACCAAACCGCTT-BHQ-1 |
| ***DCC*** | GGGTTCGGCGCGTGT | CGAAAAATACAAAAACCAACTTAAATACC | 6FAM-ACCAAAAATCGCGAACAACGACAACACT-BHQ-1 |
| ***ESR1*** | GGCGTTCGTTTTGGGATTG | GCCGACACGCGAACTCTAA | 6FAM-CGATAAAACCGAACGACCCGACGA-BHQ-1 |
| ***FLJ39739*** | GAACCCTCCGCGCCC | TGTTCGGGATGGTTGATATTTTG | 6FAM-CCGCCTAATCACCCGACCCGA-BHQ-1 |
| ***GATA5*** | AGTTACGTGATTTTGGTAGGTTTTGTT | TAATCCGAACTCCGCGCTA | 6FAM-CCCGTATCGTACGTCCTTATCGCCAAA-BHQ-1 |
| ***GDNF*** | CGGTAGTTGTCGTTGAGTCGTTC | AACAACCGCCGCTACTTTAAATA | 6FAM-CGCGCGTCGCGCTCTTAACTAAAA-BHQ-1 |
| ***HIC1*** | GTTAGGCGGTTAGGGCGTC | CCGAACGCCTCCATCGTAT | 6FAM-CAACATCGTCTACCCAACACACTCTCCTACG-BHQ-1 |
| ***HOXA1*** | TTGTTTATTAGGAAGCGGTCGTC | TCGAACCATAAAATTACAACTTTCCA | 6FAM-TCGTACGCGATCAACGCCAACAATTA-BHQ-1 |
| ***HOXA10*** | TGTATTGATGGGTTAGGAGACGTATT | CCCACCAACCACGTTAAAACA | 6FAM-CAACTCCCGACCTTCGAACCAAAATATCG-BHQ-1 |
| ***HOXA11*** | TTTTGTTTTCGATTTTAGTCGGAAT | TAATCAAATCACCGTACAAATCGAAC | 6FAM-ACCACCAAACAAACACATCCACGACTTCA-BHQ-1 |
| ***HSD17B4*** | TATCGTTGAGGTTCGACGGG | TCCAACCTTCGCATACTCACC | 6FAM-CCCGCGCCGATAACCAATACCA-BHQ-1 |
| ***ITGA4*** | TGCGGAGGCGTAGGGTC | CAACCGAAATTCCCCAACG | 6FAM-CCTACAACCGCGCGTAAACAAAAACG-BHQ-1 |
| ***MGA (I)*** | CCACCCGACTAATCATTAACTACCA | CGGCGTCGTTTCGAGTTC | 6FAM-TCGAATCACGTACTAATCGCAAACCCG-BHQ-1 |
| ***MGA (II)*** | CCACGTAAAAAAATACCGCAAAA | TCGTAGAGCGATTATCGGGTTT | 6FAM-CTTCCGAAAAAAAATCCCGACAAATCACG-BHQ-1 |
| ***MYOD1*** | GAGCGCGCGTAGTTAGCG | TCCGACACGCCCTTTCC | 6FAM-CTCCAACACCCGACTACTATATCCGCGAAA-BHQ-1 |
| ***NEUROD1*** | GTTTTTTGCGTGGGCGAAT | CCGCGCTTAACATCACTAACTAAA | 6FAM-CGCGCGACCACGACACGAAA-BHQ-1 |
| ***NEUROG1*** | CGTGTAGCGTTCGGGTATTTGTA | CGATAATTACGAACACACTCCGAAT | 6FAM-CGATAACGACCTCCCGCGAACATAAA-BHQ-1 |
| ***NUP155 (I)*** | AACGCGTACTCGACCTAAATCTTAA | CGTTTATGCGCGAATTTATATAGTTG | 6FAM-ACTTCCCGCGTCGATATCCGCTAACT-BHQ-1 |
| ***NUP155 (II)*** | AATCCGAATACATACGATCCTCTTACA | GCGGCGATGTCGGTTTT | 6FAM-ACGATCGATAAACCGTCCAACATTTTCCA-BHQ-1 |
| ***PGR*** | TTATAATTCGAGGCGGTTAGTGTTT | TCGAACTTCTACTAACTCCGTACTACGA | 6FAM-ATCATCTCCGAAAATCTCAAATCCCAATAATACG-BHQ-1 |
| ***PITX2 (I)*** | AGTTCGGTTGCGCGGTT | TACTTCCCTCCCCTACCTCGTT | 6FAM-CGACGCTCGCCCGAACGCTA-BHQ-1 |
| ***PITX2 (II)*** | GTGGTTTCGTATTAATTAGGACGTTTC | CCGACCCCGCACTACTAATAAAT | 6FAM-ACAAAACAATCCCTCCACCGCGACT-BHQ-1 |
| ***PTGS2*** | CGGAAGCGTTCGGGTAAAG | AATTCCACCGCCCCAAAC | 6FAM-TTTCCGCCAAATATCTTTTCTTCTTCGCA-BHQ-1 |
| ***SEZ6L*** | GCGTTAGTAGGGAGAGAAAACGTTC | ATACCAACCGCCTCCTCTAACC | 6FAM-CCGTCGACCCTACAAAATTTAACGCCA-BHQ-1 |
| ***SFRP1*** | CAACTCCCGACGAAACGAA | CGCGAGGGAGGCGATT | 6FAM-CACTCGTTACCACGTCCGTCACCG-BHQ-1 |
| ***SFRP2*** | AAACCTACCCGCCCGAAA | GTTGAACGGTGGTTGGAGATTC | 6FAM-CGCCTCGACGAACTTCGTTTTCCCT-BHQ-1 |
| ***SFRP4*** | TCCGCCGTCTAACACACAAA | TTCGTAATGGTCGTGGTTGGT | 6FAM-CAACGCCAACTCTCAACCTTCGAAACG-BHQ-1 |
| ***SFRP5*** | GAACGCCCCGACTAATCCTAA | TAGGCGGTCGGAGATTGGT | 6FAM-CTCCCACCTCGAAACTCCAACCCG-BHQ-1 |
| ***SIRT3*** | AACTAACACCACAAAACTAATCCGTAAC | CGTTTTTGCGTTTCGGATAAA | 6FAM-AACGCCCACACTCTTTAACGCCTCAATAA-BHQ-1 |
| ***SLIT2*** | CAATTCTAAAAACGCACGACTCTAAA | CGGGAGATCGCGAGGAT | 6FAM-CGACCTCTCCCTCGCCCTCGACT-BHQ-1 |
| ***SYK*** | GGGCGCGATATTGGGAG | GCGACTCTTCCTCATTTTAAACAAC | 6FAM-CCTTAACGCGCCCGAACAAACG-BHQ-1 |
| ***TERT*** | GGATTCGCGGGTATAGACGTT | CGAAATCCGCGCGAAA | 6FAM-CCCAATCCCTCCGCCACGTAAAA-BHQ-1 |
| ***TFF1*** | TAAGGTTACGGTGGTTATTTCGTGA | ACCTTAATCCAAATCCTACTCATATCTAAAA | 6FAM-CCCTCCCGCCAAAATAAATACTATACTCACTACAAAA-BHQ-1 |
| ***TIMP3*** | GCGTCGGAGGTTAAGGTTGTT | CTCTCCAAAATTACCGTACGCG | 6FAM-AACTCGCTCGCCCGCCGAA-BHQ-1 |
| ***TITF1*** | CGAAATAAACCGAATCCTCCTTAA | TGTTTTGTTGTTTTAGCGTTTACGT | 6FAM-CTCGCGTTTATTTTAACCCGACGCCA-BHQ-1 |
| ***TRIP10*** | CCTCCGACCACAAAACGAAT | GGTGCGGGAGTTAATGGATTT | 6FAM-AAAATTTCGATCCAACCCAAAACGCG-BHQ-1 |
| ***TWIST1*** | GTAGCGCGGCGAACGT | AAACGCAACGAATCATAACCAAC | 6FAM-CCAACGCACCCAATCGCTAAACGA-BHQ-1 |
| ***SLC6A20*** | AGGCGAATACGAATTGTAGCG | TAAAACGACGCGCCTAACG | 6FAM-CCGCGCACTAAAACTACCGTACCGAA-BHQ-1 |
| ***ZBTB16*** | ATCACGACGACAACGACAACAT | TGATTTGTTAATTTCGTAGTAGAGAGGAGTT | 6FAM-CGACAATTCGCAATACCCGCTCTCA-BHQ-1 |
| ***ZNF217 (I)*** | ACTACCTCCCGTCCACAAACAT | TGTTTCGAGTGCGGTAAAGTTTT | 6FAM-CGACCCTCCGATCCTTCTTATAAACCCTAAAATACA-BHQ-1 |
| ***ZNF217 (II)*** | CTTCTTCCCCGTTTCAAAATAAAA | TCGGTATTAGTAGCGCGTAGATAGATT | 6FAM-TCCTCCCTCGAAAACGACATTCCTCCT-BHQ-1 |

**Table S1B: General gene information.**

| **HUGO Gene Nomenclature** | **Alternative gene names** | **Chromosomal location** | **Amplicon start: location relative to transcription start (bp)** | **Amplicon end: location relative to transcription start (bp)** |
| --- | --- | --- | --- | --- |
| ***BCL2*** | Bcl-2/B-cell CLL/lymphoma2 | 18q21.3 | +209 | +292 |
| ***BRIP1 (I)*** | BRCA1 interacting protein C-terminal helicase 1; BACH1; FANCJ | 17q22-q24 | +70 | +171 |
| ***BRIP1 (II)*** | BRCA1 interacting protein C-terminal helicase 1; BACH1; FANCJ | 17q22-q24 | -256 | -154 |
| ***ZC3H4*** | Zinc finger CCCH-type containing 4 | 19q13.32 | +616 | +691 |
| ***CALCA*** | Calcitonin/Calcitonin-related polypeptide alpha; CALC1 | 11p15.2-p15.1 | -127 | -27 |
| ***CCND2*** | Cyclin D2 | [12p13](http://www.ncbi.nlm.nih.gov/mapview/maps.cgi?ORG=hum&CHR=12&maps=loc-r,morbid,gene&R1=on&query=CCND2&VERBOSE=ON&ZOOM=3) | -1079 | -1015 |
| ***CDH13*** | H-Cadherin/Cadherin 13, H-cadherin (heart)/T-cadherin; CDHH | [16q24.2-q24.3](http://www.ncbi.nlm.nih.gov/mapview/maps.cgi?ORG=hum&CHR=16&maps=loc-r,morbid,gene&R1=on&query=CDH13&VERBOSE=ON&ZOOM=3) | +140 | +242 |
| ***CDKN2C*** | Cyclin-dependent kinase inhibitor 2C (p18, inhibits CDK4) | [1p32](http://www.ncbi.nlm.nih.gov/mapview/maps.cgi?ORG=hum&CHR=1&maps=loc-r,morbid,gene&R1=on&query=CDKN2C&VERBOSE=ON&ZOOM=3) | -85 | +4 |
| ***CYP1B1*** | Cytochrome P450, family 1, Subfamily B, polypeptide 1; GLC3A; CP1B | [2p22-p21](http://www.ncbi.nlm.nih.gov/htbin-post/Omim/getmap?l601771) | -165 | -80 |
| ***DCC*** | Deleted in colorectal carcinoma; Colorectal cancer-related choromosome sequence 18 (CRC18) | [18q21.3](http://www.ncbi.nlm.nih.gov/htbin-post/Omim/getmap?l120470) | -97 | +61 |
| ***ESR1*** | Estrogen receptor receptor 1, Estrogen Receptor Alpha (ESRA); NR3A1 | 6q25.1 | +14 | +114 |
| ***FLJ39739*** | Hypothetical FLJ39739 | 1q21.1 | -493 | -414 |
| ***GATA5*** | GATA binding protein 5; bB379O24.1 | 20q13.33 | -156 | -240 |
| ***GDNF*** | Glial cell derived neurotrophic factor | [5p13.1-p12](http://www.ncbi.nlm.nih.gov/htbin-post/Omim/getmap?l600837) | -202 | -94 |
| ***HIC1*** | Hypermethylated in cancer 1; ZBTB29 | 17p13.3 | -55 | +45 |
| ***HOXA1*** | Homeobox A1; Homeobox 1F | [7p15.3](http://www.ncbi.nlm.nih.gov/htbin-post/Omim/getmap?l142955) | +151 | +233 |
| ***HOXA10*** | Homeobox A10; Homeobox 1H | [7p15-p14.2](http://www.ncbi.nlm.nih.gov/htbin-post/Omim/getmap?l142957) | -174 | -257 |
| ***HOXA11*** | Homeobox A11 | [7p15-p14.2](http://www.ncbi.nlm.nih.gov/htbin-post/Omim/getmap?l142958) | -564 | -663 |
| ***HSD17B4*** | 17beta-hydroxysteroid dehydrogenase IV | 5q2 | + 7 | +77 |
| ***ITGA4*** | Integrin alpha 4 (antigen CD49D, alpha-4 subunit of VLA-4 receptor); CD49D | 2q31-q32 | +796 | +866 |
| ***MGA (I)*** | MAX gene associated; KIAA0518; MAD5; MXD5; FLJ12634 | 15q15 | -38990 | -38892 |
| ***MGA (II)*** | MAX gene associated; KIAA0518; MAD5; MXD5; FLJ12634 | 15q15 | +1472 | +1566 |
| ***MYOD1*** | Myogenic differentiating antigen 1; Myogenic determining factor 3/MYF3/PUM | 11p15.4 | -375 | -302 |
| ***NEUROD1*** | Neurogenic differentiation 1; B-cell E-box transctivator 2 (BETA2); BHF-1 | 2q32 | +35 | -47 |
| ***NEUROG1*** | Neurogenin 1; NEOROD3; AKA | 5q23-q31 | -132 | -220 |
| ***NUP155 (I)*** | Nucleoporin 155kDa, KIAA0791, N155 | 5p13 | -432 | -353 |
| ***NUP155 (II)*** | nucleoporin 155kDa, KIAA0791, N155 | 5p13 | +185 | +244 |
| ***PGR*** | Progesterone receptor B | 11q22 | +99 | +192 |
| ***PITX2 (I)*** | Paired-like homeodomain transcription factor 2; Pituitary homeobox 2 (PTX2) | [4q25-26](http://www.ncbi.nlm.nih.gov/htbin-post/Omim/getmap?l601542) | +4818 | +4716 |
| ***PITX2 (II)*** | Paired-like homeodomain transcription factor 2; Pituitary homeobox 2 (PTX2) | [4q25-26](http://www.ncbi.nlm.nih.gov/htbin-post/Omim/getmap?l601542) | +113 | +192 |
| ***PTGS2*** | Prostaglandin-endoperoxide synthase 2; COX2; GRIPGHS | 1q25.2-q25.3 | -362 | -217 |
| ***SEZ6L*** | Seizure related 6 homolog (mouse)-like; KIAA0927 | [22q12.1](http://www.ncbi.nlm.nih.gov/htbin-post/Omim/getmap?l607021) | -300 | -198 |
| ***SFRP1*** | Secreted frizzled-related protein 1; FRP; FRP1; FrzA; SARP2 | [8p12-p11.1](http://www.ncbi.nlm.nih.gov/htbin-post/Omim/getmap?l604156) | -130 | -58 |
| ***SFRP2*** | Secreted frizzled-related protein 2; FRP-2; SARP1; SDF-5 | 4q31.3 | -599 | -533 |
| ***SFRP4*** | Secreted frizzled-related protein 4; FRPHE | 7p14-p13 | -40 | +64 |
| ***SFRP5*** | Secreted frizzled-related protein 5; SARP3 | 10q24.1 | -59 | +27 |
| ***SIRT3*** | Sirtuin (silent mating type information regulation 2 homolog) 3 (S. cerevisiae); SIR2L3 | 11p15.5 | -526 | -392 |
| ***SLIT2*** | FLJ14420; SLIL3 | 4p15.2 | -390 | -489 |
| ***SYK*** | Spleen tyrosine kinase/LKB1 | [9q22](http://www.ncbi.nlm.nih.gov/htbin-post/Omim/getmap?l600085) | -65 | +11 |
| ***TERT*** | Telomerase reverse transcriptase; TRT; TP2; TCS1; EST2 | [5p15.33](http://www.ncbi.nlm.nih.gov/mapview/maps.cgi?ORG=hum&CHR=5&maps=loc-r,morbid,gene&R1=on&query=TERT&VERBOSE=ON&ZOOM=3) | -279 | -163 |
| ***TFF1*** | Trefoil factor 1/pS2 (breast cancer, estrogen-inducible sequence); BCE1; D21S21 | 21q22.3 | -458 | -311 |
| ***TIMP3*** | Tissue Inhibitor of metallinoproteinase 3; SFD | [22q12.1-q13.2](http://www.ncbi.nlm.nih.gov/mapview/maps.cgi?ORG=hum&CHR=22&maps=loc-r,morbid,gene&R1=on&query=TIMP3&VERBOSE=ON&ZOOM=3) | +1056 | +1150 |
| ***TITF1*** | Thyroid transcription factor 1; NKX2A; TEBP; BCH; TTF-1 | [14q13](http://www.ncbi.nlm.nih.gov/htbin-post/Omim/getmap?l600635) | -74 | +54 |
| ***TRIP10*** | Thyroid hormone receptor interactor 10; STOT; STP; HSTP; CIP4 | 19p13.3 | -550 | -467 |
| ***TWIST1*** | Twist homolog (acrocephalosyndactyly 3; Saethre-Chotzen syndrome) (Drosophila) | [7p21](http://www.ncbi.nlm.nih.gov/mapview/maps.cgi?ORG=hum&CHR=7&maps=loc-r,morbid,gene&R1=on&query=TWIST1&VERBOSE=ON&ZOOM=3) | +669 | +745 |
| ***SLC6A20*** | XT3/X transporter protein 3; Solute carrier family 6 (neurotransmitter transporter) member 20, SIT1, XTRP3 | [3p21.3](http://www.ncbi.nlm.nih.gov/mapview/maps.cgi?ORG=hum&CHR=3&maps=loc-r,morbid,gene&R1=on&query=SLC6A20&VERBOSE=ON&ZOOM=3) | +326 | +192 |
| ***ZBTB16*** | Promyelocytic leukemia zinc finger protein (PLZF); ZNF145 | 11q23.2 | -32 | +55 |
| ***ZNF217 (I)*** | Zinc finger protein 217; ZABC1 | 20q13.2 | +1398 | +1519 |
| ***ZNF217 (II)*** | Zinc finger protein 217; ZABC1 | 20q13.2 | +1000 | +1116 |
