# Supplementary material for: Epigenotyping in Peripheral Blood Cell DNA and Breast Cancer Risk: A Proof of Principle Study
Source: PLoS One. 2008 Jul 16;3(7):e2656. doi: 10.1371/journal.pone.0002656 (PMC2442168; doi:10.1371/journal.pone.0002656)
Supplement: Table S2 — Clinicopathological characteristics of breast cancer cases used for the study. UICC = Union internationale contre le cancer; *Among those with information; n/a = not applicable. (0.05 MB DOC) [file pone.0002656.s002.doc]

| **TUMOR CHARACTERISTICS** | | ***n*** | **%*** |
| --- | --- | --- | --- |
|  |  |  |  |
| **Histology** | Lobular | 61 | 18.3 |
|  | Ductal | 236 | 70.9 |
|  | Other | 36 | 10.8 |
|  | Unknown | 20 | n/a |
|  |  |  |  |
| **UICC stage** | I | 118 | 34.6 |
|  | II | 163 | 48.9 |
|  | III | 45 | 13.5 |
|  | IV | 15 | 4.5 |
|  | Unknown | 12 | n/a |
|  |  |  |  |
| **Grading** | 1 | 29 | 8.5 |
|  | 2 | 175 | 51.2 |
|  | 3 | 138 | 40.4 |
|  | Unknown | 11 | n/a |
|  |  |  |  |
| **Estrogen Receptor (ER)** | Positive | 231 | 77.3 |
|  | Negative | 68 | 22.7 |
|  | Unknown | 54 | n/a |
|  |  |  |  |
| **Progesterone Receptor (PR)** | Positive | 193 | 65.9 |
|  | Negative | 100 | 34.1 |
|  | Unknown | 60 | n/a |
|  |  |  |  |
| **HER2** | Positive | 51 | 27.3 |
|  | Negative | 136 | 72.7 |
|  | Unknown | 166 | n/a |
|  |  |  |  |
