# Supplementary material for: Epigenotyping in Peripheral Blood Cell DNA and Breast Cancer Risk: A Proof of Principle Study
Source: PLoS One. 2008 Jul 16;3(7):e2656. doi: 10.1371/journal.pone.0002656 (PMC2442168; doi:10.1371/journal.pone.0002656)
Supplement: Table S3 — Mean and median percentage of methylated reference (PMR) value of the 25 different loci. 7 genes of those analyzed demonstrated significant differences in the level of methylation between cases and controls, based on the quantitative results on peripheral blood cell DNA. ERT = estrogen receptor-α target; DMHR = differently methylated depending on hormone receptor status; PCGT = stem cell polycomb group target; MBC = methylated in breast cancer; * indicates P-values <0.05 (0.08 MB DOC) [file pone.0002656.s003.doc]

| **Gene group** | **Gene loci** | **Cases** | | |  | **Controls** | | |  | ***P*-value** |
| --- | --- | --- | --- | --- | --- | --- | --- | --- | --- | --- |
|  |  | *n* | mean | median |  | *n* | mean | median |  |  |
| **ERT** | *BRIP1 (I)* | 307 | 0.0644 | 0 |  | 653 | 0.0534 | 0 |  | 0.934 |
|  | *ESR1* | 321 | 0.0378 | 0 |  | 676 | 0.0853 | 0 |  | 0.559 |
|  | *SIRT3* | 295 | 0.0302 | 0 |  | 627 | 0.0285 | 0 |  | 0.078 |
|  | ***NUP155 (I)*** | **307** | **0.0862** | **0** |  | **653** | **0.1421** | **0** |  | **0.037*** |
|  | *PITX2 (I)* | 321 | 0.2732 | 0 |  | 676 | 0.4211 | 0 |  | 0.158 |
|  | *PITX2 (II)* | 321 | 0.1938 | 0 |  | 676 | 0.2062 | 0 |  | 0.923 |
|  | ***DCC*** | **303** | **0.121** | **0** |  | **638** | **0.1625** | **0** |  | **0.049*** |
|  | ***ZNF217 (II)*** | **303** | **0.3444** | **0** |  | **638** | **0.4804** | **0** |  | **0.001*** |
|  | *FLJ39739* | 295 | 0.0198 | 0.0001 |  | 627 | 0.0083 | 0 |  | 0.279 |
|  | *PGR* | 321 | 0.5829 | 0.1525 |  | 676 | 0.7026 | 0.1956 |  | 0.164 |
| **DMHR** | *TIMP3* | 321 | 0.0542 | 0 |  | 676 | 0.1085 | 0 |  | 0.373 |
|  | *CDH13* | 321 | 0.0423 | 0 |  | 676 | 0.0889 | 0 |  | 0.459 |
|  | *HSD17B4* | 321 | 0.0309 | 0 |  | 676 | 0.0138 | 0 |  | 0.148 |
|  | ***PTGS2*** | **321** | **0.5563** | **0.0882** |  | **676** | **0.6766** | **0.1929** |  | **0.007*** |
| **PCGT** | *SLC6A20* | 321 | 0.0069 | 0 |  | 676 | 0.0123 | 0 |  | 0.308 |
|  | *NEUROG1* | 303 | 0.0123 | 0 |  | 638 | 0.0121 | 0 |  | 0.794 |
|  | *HOXA1* | 307 | 0.0491 | 0 |  | 653 | 0.0597 | 0 |  | 0.221 |
|  | ***TITF1*** | **321** | **0.0516** | **0** |  | **676** | **0.1186** | **0** |  | **0.019*** |
|  | *GDNF* | 307 | 0.1131 | 0 |  | 653 | 0.2128 | 0 |  | 0.136 |
|  | ***NEUROD1*** | **299** | **0.0999** | **0** |  | **642** | **0.1946** | **0** |  | **0.007*** |
|  | ***SFRP1*** | **321** | **0.2049** | **0** |  | **676** | **0.3259** | **0** |  | **0.013*** |
|  | *MYOD1* | 321 | 0.2803 | 0.0055 |  | 676 | 0.3667 | 0.0176 |  | 0.102 |
| **MBC** | *SYK* | 321 | 0.0023 | 0 |  | 676 | 0.0007 | 0 |  | 0.858 |
|  | *CYP1B1* | 321 | 0.0077 | 0 |  | 676 | 0.034 | 0 |  | 0.106 |
|  | *SEZ6L* | 307 | 0.0949 | 0 |  | 653 | 0.11 | 0.0005 |  | 0.131 |
