# Supplementary material for: Epigenotyping in Peripheral Blood Cell DNA and Breast Cancer Risk: A Proof of Principle Study
Source: PLoS One. 2008 Jul 16;3(7):e2656. doi: 10.1371/journal.pone.0002656 (PMC2442168; doi:10.1371/journal.pone.0002656)
Supplement: Table S4 — Odds ratios for invasive ductal and lobular breast cancer risk associated with peripheral blood DNA methylation. S4A: Risk for invasive ductal breast cancer. S4B: Risk for invasive lobular breast cancer. (0.14 MB DOC) [file pone.0002656.s004.doc]

**Table S4**

**Table S4A. Odds ratios for invasive ductal breast cancer risk associated with peripheral blood DNA methylation.** ERT=estrogen receptor-α target; DMHR=differently methylated depending on hormone receptor status; PCGT= stem cell polycomb group target; MBC=methylated in breast cancer; OR=odds ratio; CI=confidence interval;* indicates *P* values < 0.05.

| **Gene Group** | **Gene Loci** | **Positive invasive ductal Cases** | **Positive Controls** |  | **Adjusted for age** | |  | **Adjusted for age and family history** | |
| --- | --- | --- | --- | --- | --- | --- | --- | --- | --- |
|  |  |  |  |  | **OR (95% CI)** | **p value** |  | **OR (95% CI)** | **p value** |
| **ERT** | *BRIP1 (I)* | 29,8%(61/205) | 30,5%(199/653) |  | 1,05(0,74-1,48) | 0,797 |  | 1,04(0,74-1,47) | 0.814 |
|  | *ESR1* | 14%(30/215) | 13,5%(91/676) |  | 0,93(0,6-1,46) | 0,764 |  | 0,93(0,59-1,45) | 0.736 |
|  | *SIRT3* | 19,8%(39/197) | 16,3%(102/627) |  | 0,79(0,52-1,19) | 0,262 |  | 0,79(0,52-1,19) | 0.258 |
|  | *NUP155 (I)* | 22%(45/205) | 28,6%(187/653) |  | 1,42(0,98-2,06) | 0,067 |  | 1,41(0,97-2,05) | 0.07 |
|  | *PITX2 (I)* | 34,4%(74/215) | 38%(257/676) |  | 1,16(0,84-1,6) | 0,373 |  | 1,15(0,83-1,59) | 0.4 |
|  | *PITX2 (II)* | 50,2%(108/215) | 48,5%(328/676) |  | 0,94(0,69-1,28) | 0,69 |  | 0,94(0,69-1,28) | 0.691 |
|  | *DCC* | 34,7%(70/202) | 41,7%(266/638) |  | 1,33(0,96-1,86) | 0,09 |  | 1,31(0,94-1,83) | 0.108 |
|  | ***ZNF217 (II)*** | **40,6%(82/202)** | **48,9%(312/638)** |  | **1,4(1,01-1,94)** | **0,04*** |  | **1,4(1,02-1,94)** | **0,04*** |
|  | *FLJ39739* | 52,3%(103/197) | 49,4%(310/627) |  | 0,87(0,63-1,2) | 0,395 |  | 0,87(0,63-1,2) | 0.388 |
|  | *PGR* | 73%(157/215) | 70,7%(478/676) |  | 0,9(0,64-1,27) | 0,55 |  | 0,9(0,64-1,27) | 0.549 |
| **DMHR** | *TIMP3* | 12,6%(27/215) | 14,2%(96/676) |  | 1,15(0,73-1,83) | 0,544 |  | 1,15(0,73-1,82) | 0.555 |
|  | *CDH13* | 13,5%(29/215) | 15,4%(104/676) |  | 1,17(0,75-1,83) | 0,478 |  | 1,18(0,76-1,84) | 0.465 |
|  | *HSD17B4* | 18,6%(40/215) | 14,3%(97/676) |  | 0,73(0,49-1,1) | 0,131 |  | 0,74(0,49-1,11) | 0.141 |
|  | *PTGS2* | 72,6%(156/215) | 77,1%(521/676) |  | 1,26(0,89-1,79) | 0,199 |  | 1,24(0,87-1,76) | 0.231 |
| **PCGT** | *SLC6A20* | 1,4%(3/215) | 1,8%(12/676) |  | 1,31(0,37-4,7) | 0,677 |  | 1,41(0,39-5,12) | 0.598 |
|  | *NEUROG1* | 5%(10/202) | 4,7%(30/638) |  | 0,96(0,46-2,02) | 0,923 |  | 0,96(0,46-2,01) | 0.911 |
|  | *HOXA1* | 12,2%(25/205) | 13,9%(91/653) |  | 1,13(0,7-1,82) | 0,609 |  | 1,12(0,7-1,81) | 0.637 |
|  | *TITF1* | 15,3%(33/215) | 19,5%(132/676) |  | 1,33(0,88-2,03) | 0,18 |  | 1,33(0,88-2,03) | 0.18 |
|  | *GDNF* | 14,1%(29/205) | 18,7%(122/653) |  | 1,36(0,87-2,12) | 0,174 |  | 1,35(0,87-2,11) | 0.179 |
|  | ***NEUROD1*** | **30,5%(61/200)** | **38,9%(250/642)** |  | **1,44(1,02-2,03)** | **0,038*** |  | **1,46(1,03-2,05)** | **0,032*** |
|  | ***SFRP1*** | **29,3%(63/215)** | **37,4%(253/676)** |  | **1,43(1,02-2)** | **0,037*** |  | **1,43(1,02-2)** | **0,036*** |
|  | *MYOD1* | 63,7%(137/215) | 63,5%(429/676) |  | 0,99(0,72-1,37) | 0,964 |  | 0,99(0,72-1,37) | 0.95 |
| **MBC** | *SYK* | 0,9%(2/215) | 2,4%(16/676) |  | 2,58(0,59-11,34) | 0,21 |  | 2,61(0,59-11,5) | 0.204 |
|  | *CYP1B1* | 7,4%(16/215) | 4,9%(33/676) |  | 0,64(0,34-1,19) | 0,16 |  | 0,64(0,34-1,19) | 0.157 |
|  | *SEZ6L* | 52,7%(108/205) | 52,8%(345/653) |  | 0,99(0,72-1,36) | 0,953 |  | 0,99(0,72-1,35) | 0.935 |

**Table S4B. Odds ratios for invasive lobular breast cancer risk associated with peripheral blood DNA methylation.** ERT=estrogen receptor-α target; DMHR=differently methylated depending on hormone receptor status; PCGT= stem cell polycomb group target; MBC=methylated in breast cancer; OR=odds ratio; CI=confidence interval;* indicates *P* values < 0.05

| **Gene Group** | **Gene Loci** | **Positive invasive lobular Cases** | **Positive Controls** |  | **Adjusted for age** | |  | **Adjusted for age and family history** | |
| --- | --- | --- | --- | --- | --- | --- | --- | --- | --- |
|  |  |  |  |  | **OR (95% CI)** | **p value** |  | **OR (95% CI)** | **p value** |
| **ERT** | *BRIP1 (I)* | 23,6%(13/55) | 30,5%(199/653) |  | 1,38(0,72-2,64) | 0,325 |  | 1,39(0,72-2,67) | 0.326 |
|  | *ESR1* | 8,8%(5/57) | 13,5%(91/676) |  | 1,64(0,64-4,24) | 0,306 |  | 1,7(0,65-4,44) | 0.279 |
|  | *SIRT3* | 11,3%(6/53) | 16,3%(102/627) |  | 1,61(0,67-3,89) | 0,289 |  | 1,61(0,66-3,93) | 0.293 |
|  | *NUP155 (I)* | 20%(11/55) | 28,6%(187/653) |  | 1,64(0,82-3,25) | 0,159 |  | 1,64(0,82-3,28) | 0.162 |
|  | ***PITX2 (I)*** | **26,3%(15/57)** | **38%(257/676)** |  | **1,86(1,01-3,44)** | **0,048*** |  | 1,76(0,94-3,28) | 0.076 |
|  | *PITX2 (II)* | 50,9%(29/57) | 48,5%(328/676) |  | 0,91(0,53-1,56) | 0,725 |  | 0,87(0,5-1,51) | 0.613 |
|  | ***DCC*** | **27,3%(15/55)** | **41,7%(266/638)** |  | **1,98(1,07-3,67)** | **0,03*** |  | 1,82(0,98-3,41) | 0.059 |
|  | *ZNF217 (II)* | 40%(22/55) | 48,9%(312/638) |  | 1,45(0,83-2,55) | 0,195 |  | 1,45(0,82-2,56) | 0.204 |
|  | *FLJ39739* | 43,4%(23/53) | 49,4%(310/627) |  | 1,37(0,77-2,43) | 0,28 |  | 1,39(0,78-2,48) | 0.268 |
|  | *PGR* | 63,2%(36/57) | 70,7%(478/676) |  | 1,44(0,81-2,53) | 0,211 |  | 1,36(0,76-2,41) | 0.299 |
| **DMHR** | *TIMP3* | 8,8%(5/57) | 14,2%(96/676) |  | 1,69(0,65-4,35) | 0,279 |  | 1,73(0,67-4,52) | 0.259 |
|  | *CDH13* | 8,8%(5/57) | 15,4%(104/676) |  | 1,86(0,72-4,77) | 0,199 |  | 1,91(0,74-4,94) | 0.185 |
|  | *HSD17B4* | 12,3%(7/57) | 14,3%(97/676) |  | 1,22(0,54-2,78) | 0,632 |  | 1,15(0,5-2,64) | 0.742 |
|  | *PTGS2* | 71,9%(41/57) | 77,1%(521/676) |  | 1,32(0,72-2,42) | 0,372 |  | 1,23(0,67-2,29) | 0.506 |
| **PCGT** | *SLC6A20* | 0%(0/57) | 1,8%(12/676) |  |  |  |  |  |  |
|  | *NEUROG1* | 1,8%(1/55) | 4,7%(30/638) |  | 2,67(0,36-20,07) | 0,339 |  | 2,61(0,34-19,84) | 0.355 |
|  | *HOXA1* | 9,1%(5/55) | 13,9%(91/653) |  | 1,7(0,66-4,39) | 0,275 |  | 1,68(0,65-4,38) | 0.287 |
|  | ***TITF1*** | **8,8%(5/57)** | **19,5%(132/676)** |  | **2,65(1,04-6,79)** | **0,042*** |  | **2,65(1,03-6,83)** | **0,043*** |
|  | ***GDNF*** | **7,3%(4/55)** | **18,7%(122/653)** |  | **3,04(1,07-8,6)** | **0,036*** |  | 2,8(0,98-7,96) | 0.054 |
|  | *NEUROD1* | 28,3%(15/53) | 38,9%(250/642) |  | 1,66(0,89-3,09) | 0,111 |  | 1,68(0,89-3,14) | 0.107 |
|  | *SFRP1* | 31,6%(18/57) | 37,4%(253/676) |  | 1,36(0,76-2,44) | 0,299 |  | 1,4(0,78-2,54) | 0.261 |
|  | ***MYOD1*** | **49,1%(28/57)** | **63,5%(429/676)** |  | **1,85(1,07-3,2)** | **0,027*** |  | **1,81(1,04-3,14)** | **0,037*** |
| **MBC** | *SYK* | 5,3%(3/57) | 2,4%(16/676) |  | 0,46(0,13-1,63) | 0,226 |  | 0,41(0,11-1,48) | 0.172 |
|  | *CYP1B1* | 8,8%(5/57) | 4,9%(33/676) |  | 0,55(0,2-1,48) | 0,238 |  | 0,56(0,2-1,55) | 0.265 |
|  | *SEZ6L* | 41,8%(23/55) | 52,8%(345/653) |  | 1,61(0,92-2,83) | 0,095 |  | 1,51(0,86-2,67) | 0.152 |
